# Building evidences in Public Health Emergency Preparedness (“BePHEP” Project)—a systematic review

**DOI:** 10.1186/s12939-025-02382-w

**Published:** 2025-02-11

**Authors:** Michelangelo Mercogliano, Gloria Spatari, Chiara Noviello, Francesca Di Serafino, Maria Elisabetta Mormile, Giuseppa Granvillano, Annalisa Iagnemma, Riccardo Mimmo, Irene Schenone, Eleonora Raso, Andrea Sanna, Enrica Frasson, Veronica Gallinoro, Marcello Di Pumpo, Duha Shellah, Caterina Rizzo, Nunzio Zotti

**Affiliations:** 1 Department of Public Health, University “Federico II” of Naples, Via Sergio Pansini 5, Naples, 80131 Italy; 2 Department of Health Sciences, University of Genoa, Genoa, 16132 Italy; 3 Interdisciplinary Department of Medicine, Aldo Moro University of Bari, Bari, 70121 Italy; 4 Department of Translational Research and of New Surgical and Medical Technologies, University of Pisa, Pisa, Italy; 5 Local Health Authority ASL LE, Via miglietta, 5, Lecce, 73100 Italy; 6 Department of Medical and Surgical Sciences and Advanced Technologies “GF Ingrassia”, University of Catania, Catania, 95123 Italy; 7 University Hospital Policlinico “G. Rodolico-San Marco”, Via Santa Sofia 78, Catania, 95124 Italy; 8 Department of Life, Health and Environmental Sciences-University of L’Aquila, L’Aquila, 67100 Italy; 9 Department of Public Health Sciences, University of Turin, Turin, Italy; 10 Regional Health Agency of Liguria (A.Li.Sa.), Genoa, 16121 Italy; 11 School of Public Health, Department of Public Health, Experimental and Forensic Medicine, University of Pavia, Pavia, 27100 Italy; 12 Post Graduate School of Public Health, University of Siena, Siena, Italy; 13 Department of Cardiac, Thoracic and Vascular Sciences and Public Health, University of Padua, Padua, 35128 Italy; 14 Department of Health Sciences, University of Florence, Florence, 50134 Italy; 15 Section of Hygiene, University Department of Life Sciences and Public Health, Università Cattolica del Sacro Cuore, Rome, 00168 Italy; 16 Euganea Local Health Authority, Veneto Region, AULSS6 Padua, Italy; 17 Medical and Health Sciences Division and Women in Global Health, Palestine, Academy for Science and Technology, Ramalla, Palestine; 18 Faculty of Medicine, Arab American University, Jenin, Palestine; 19 EMR Youth Council, World Health Organization, Geneva, Switzerland

**Keywords:** Health Emergency Preparedness, Humanitarian crises, Low- and Middle-Income Countries, LMICs, Infectious diseases, Communicable diseases, Health system resilience, Natural disasters, Armed conflicts, Systematic review

## Abstract

**Introduction:**

Humanitarian crises exacerbate the vulnerability of already fragile healthcare systems and significantly increase the risk of infectious disease outbreaks in low- and middle-income countries (LMICs). This systematic review aims to evaluate strategies and interventions implemented in LMICs to prevent and manage infectious diseases outbreaks during humanitarian crises from 2018 to 2023.

**Methods:**

A comprehensive literature search was conducted across Scopus, PubMed, and Web of Science, adhering to the PRISMA guideline and the SPIDER framework to identify relevant studies. The review included studies published between 2018 and 2023 focusing on infectious disease prevention and management in LMICs during humanitarian crises. Study quality was assessed using the Joanna Briggs Institute checklist.

**Results:**

Eleven studies were identified from 1,415 unique articles. These studies addressed diverse interventions, including vaccination campaigns, epidemiologic surveillance, and integrated health services. Cholera outbreaks in Haiti and Mozambique, triggered by gang violence, internal migration, and Cyclone Kenneth, were addressed through epidemiological surveillance, case management, WASH (Water, Sanitation, and Hygiene) service improvements, and oral vaccination campaigns. Mathematical models guided cholera vaccination in Thailand's refugee camps. In India, surveillance and rapid response measures successfully prevented infectious disease outbreaks during the Kumbh Mela gathering. The Philippines improved response times to climate-related disasters using point-of-care testing and spatial care pathways. Despite challenges in Yemen, evaluating malaria surveillance systems led to recommendations for integrating multiple systems. Uganda developed a national multi-hazard emergency plan incorporating vaccination, communication, and risk management, proving useful during the refugee crisis and Ebola outbreak. In South Sudan, integrating immunisation services into nutrition centres increased vaccination coverage among children. Nigeria experienced a rise in measles cases during armed conflicts despite vaccination efforts, while visual communication strategies improved SARS-CoV-2 vaccination rates.

**Conclusion:**

These interventions highlight the importance of multimodal, targeted, and collaborative responses to address complex health crises without relying on unsustainable investments. Despite the effectiveness of these interventions, infrastructure limitations, insecurity, and logistical constraints were noted. These findings emphasize the need for adaptable and resilient healthcare systems and international collaboration to safeguard the right to health during complex humanitarian crises.

**Supplementary Information:**

The online version contains supplementary material available at 10.1186/s12939-025-02382-w.

## Introduction

In 2023, 399 natural disasters caused 86,000 deaths and affected 93 million people, with 85% of the impact occurring in Africa and Asia [1]. Many low- and middle-income countries (LMICs) [2] are particularly vulnerable to humanitarian crises due to specific socioeconomic, political, and geographical conditions. These crises exacerbate political instability, violence, and conflict, leading to increased displacement, limited healthcare access, and overcrowded shelters [3]. Health risks in these regions include heightened epidemic risks [4], including vaccine-preventable diseases such as measles and polio, as well as more complex infections such as tuberculosis and malaria [5, 6], water contamination, and malnutrition [7].

A 2023 systematic review [8] revealed that natural disasters significantly increase the risk of infectious disease outbreaks due to infrastructure damage, displacement, and environmental changes. Floods and hurricanes, for example, are linked to vector-borne diseases such as malaria and dengue by creating mosquito breeding sites and the collapse of health and sanitation systems can worsen the situation. Displacement and migration spread infections of new pathogens or carried diseases to new areas. Natural disasters increase the risk of infectious diseases through environmental changes and weakened health infrastructure. In the same way, conflicts and political instability trigger humanitarian crises that spread infectious diseases by displacing populations and limiting healthcare access. These crises disrupt supply chains, cause shortages of medications and healthcare workers, and impair vaccination programs [9]. The COVID-19 pandemic has highlighted these vulnerabilities, demonstrating how infectious diseases can rapidly destabilise entire healthcare systems and communities [10, 11].

Poor sanitation, overcrowding, contaminated water, and inadequate waste management increase the risk of outbreaks, including diarrhoea, which is a leading cause of death in LMICs, both under normal conditions and during disasters [12–14]. Infrastructure collapse and disrupted health services reduce immunisation, leading to preventable disease resurgence, whereas displaced populations spread infections [15–18].

Humanitarian crises, whether due to conflict, natural disasters, or health emergencies, severely weaken the health infrastructure and response capacity of LMICs [19]. Primary defences against infection involve environmental hygiene to maintain sanitary conditions and curb disease spread [20]. A recent scoping review of nonpharmacological interventions (NPIs) in crisis-affected populations emphasised the need for context specific, effective, and sustainable interventions [21]. Key measures include vector control, regular cleaning, disinfection, waste management, public education, clean water access, improved sanitation, and travel restrictions. Surveillance and response measures, such as active case detection, contact tracing, isolation, and quarantine, are crucial for early containment. Individual measures such as the use of insecticide-treated nets, hand hygiene, water purification, condom distribution, and mask use are essential for reducing transmission and strengthening health system responses [21]. NPIs, as social distancing and proactive risk communicators, are vital at both the individual and community levels [22], and vaccination campaigns must continue to maintain immunisation coverage and address emerging threats [21]. In those situations, international collaboration can support knowledge sharing, capacity building, joint research, equitable resource distribution, and unified protocols, enhancing disease prevention, surveillance, and control [23].

This systematic review aims to analyse the strategies and interventions implemented in LMICs to counter the spread of infectious diseases during newly emerging humanitarian crises.

## Methods

This systematic review was conducted following the PRISMA guidelines [24]. The inclusion and exclusion criteria were assessed via the SPIDER framework [25].

### Eligibility Criteria

Inclusion and exclusion criteria are summarised in Table 1. These criteria included studies (e.g. original research, case studies, report or communicating) being conducted in LMICs, in accordance with the World Bank Group [2]. The focus was to analyse how LMICs, during a humanitarian crisis, face the spread of an infectious disease by implementing prevention and/or response systems for epidemics and achieving positive health outcomes. Additionally, we included studies focusing on preparedness (improved resilience and sustainability of the national health system in the face of large-scale epidemics, reduced deaths and disease prevalence). Types of scientific research, such as systematic reviews and meta-analyses, or studies in languages other than English or without available full text were excluded.

**Table 1:** Eligibility criteria

Criteria	Inclusion	Exclusion
Language	English	Not English
Geographical area of interest	Worldwide Low-income and middle-income countries (LMICs)	Without a single national setting Not LMICs
Timeframe	Published between 2018 and 2023	Published before 2018 Published after 2023
(S) Sample	People facing humanitarian crisis as conflict zones, people forced to emigrate, areas with a high impact of global warming, et similia	Population not in a complex setting with humanitarian crisis
(PI) Phenomenon of Interest	A real or a simulated infectious disease spread	Any not-infectious disease spread, routinary infectious disease not linked with humanitarian crisis
(D) Design	Quantitative or qualitative original research Cross-sectional studies / comparative cross-sectional studies / RCTs, non-RCTs Case study, report, communication Observational, mixed methods, cross-sectional, trial	Book reviews, corrigendum articles, and theoretical and critical reviews Systematic review, metanalysis Book chapter, opinion, editorial opinion-based studies, letter to editors, study protocols
(E) Evaluation	Intervention and preparedness and positive health outcomes (improved resilience and sustainability of the national health system in the face of large-scale epidemics, reduced deaths and disease prevalence)	No intervention, no evaluation, nor real effectiveness data or theoretical data or possible results
(R) Research type	Qualitative studies, quantitative studies, and mixed-method studies Peer-reviewed published literature	Grey literature, dissertations, letters, editorials, theses, dissertations, and conference proceedings without peer-review process

### Search strategy

Articles were selected from the Scopus, PubMed, and Web of Science databases. The team agreed on the research string to ensure comprehensive literature coverage. The keywords used are listed in the ***supplementary material***. Additional relevant papers were manually searched from reference lists of collected studies and reviews.

### Data extraction and quality assessment

Twelve reviewers screened the articles to identify those meeting the inclusion criteria. Duplicate entries were removed. The reviewers utilised the Rayyan web-based application as task management tool [26]. Full-text reviews were conducted even when the abstract lacked sufficient information. Data extraction of included studies was conducted by two reviewers independently. Senior reviewer resolved any disagreements.

The quality of the studies was assessed via the Joanna Briggs Institute (JBI) Critical Appraisal tools, with specific checklists applied according to the type of study, ensuring a tailored and appropriate evaluation for each study type [27]. This evaluation of studies’ quality allows to inform the synthesis of the extracted evidence. Two reviewers independently assessed each study, and conflicts and uncertainties were resolved through discussions with the senior reviewer. Scores were converted to percentages to facilitate the quality rating.

## Results

### Study selection

**Fig. 1:**
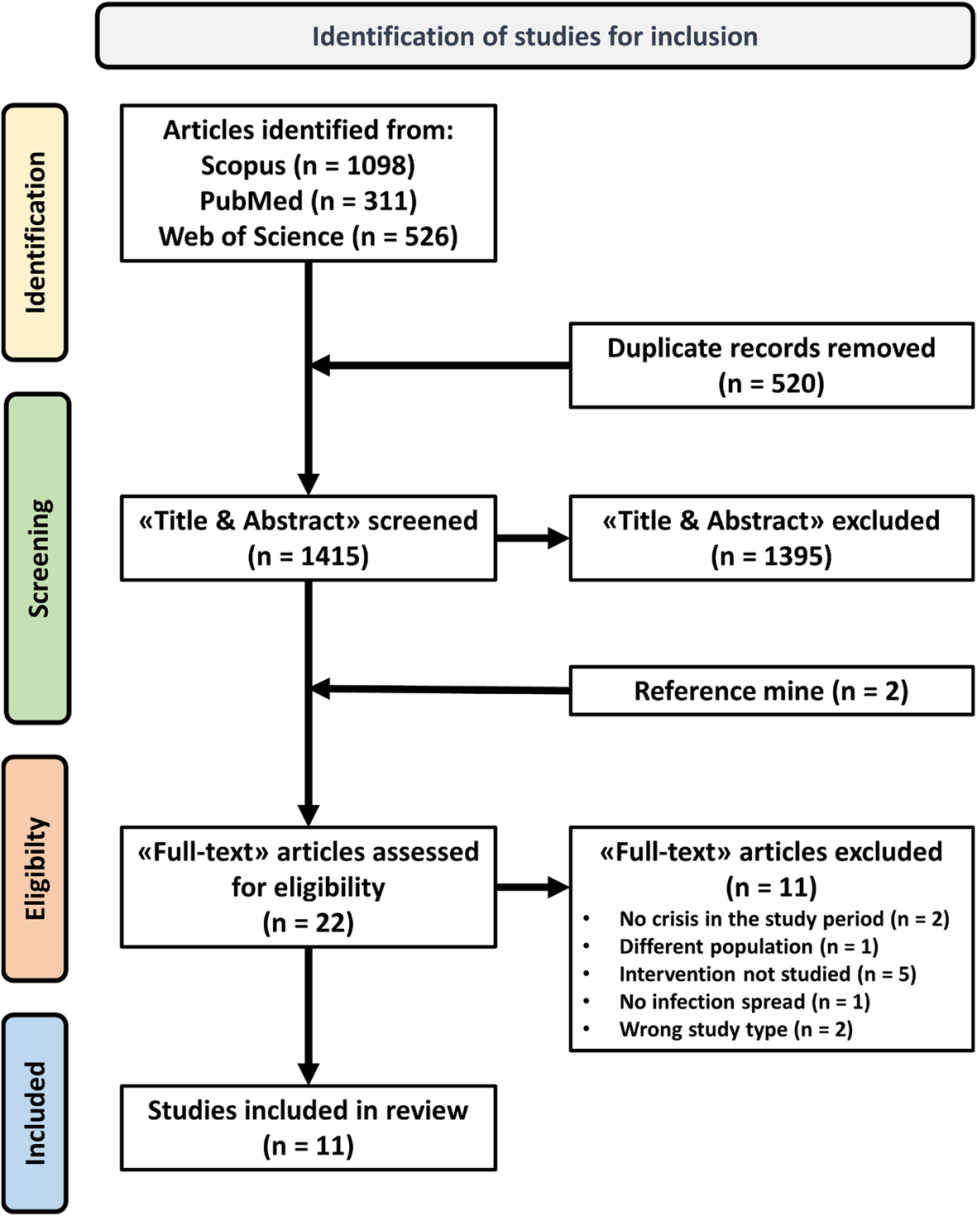
PRISMA flow diagram of the literature search, abstract screening, full article assessment for exclusion and inclusion criteria, with the most common reasons for exclusion being detailed

After removing duplicates, the initial pool of 1935 studies was reduced to 1415 unique studies. Upon review of “titles and abstracts”, 1395 studies were excluded. Among 22 studies, 11 met the inclusion criteria and were selected. Among these, 2 articles [28, 29] were retrieved from 1 article [30]. The other 11 studies were excluded for the following reasons: 2 studies did not have a setting during the crisis period, 1 study considered animals as the population, 5 studies did not describe an intervention, 1 study did not have any infection spread, and 2 studies were excluded on the basis of study design. A visual representation with more information on the selection process is provided in the PRISMA diagram (Fig. 1).

### Quality assessment

The majority of the studies achieved a score of 100% on the JBI Critical Appraisal tools checklist, the lowest score was 71.3%. The score for each study is reported in Table 2; for further details on the critical appraisal, please refer to the supplementary materials.

**Table 2:** Studies characteristics and results

**Article**	**Category**	**Setting**				**Crisis**		**Infectious Disease**
		***Country***	***Study Period***	***Setting of the Study***	***Involved Population***	***Humanitarian Crisis***	***Impact of the Crisis***	
[31]	Containing community and communicable infectious diseases	Haiti	September 2022–January 2023	Metropolitan area of Port-au-Prince, Haiti (primarily Ouest Department), with extension to other departments	General population in affected areas, including children under 10 years old	Non-state armed groups, gang violence, social unrest, population displacement, and fuel shortages	The destruction and impairment of public health infrastructure, sanitation services, and healthcare delivery systems, exacerbated by social unrest and insecurity, have led to increased cholera cases and limited access to safe water, sanitation, and hygiene (WASH) services	Cholera
[32]	Containing community and communicable infectious diseases	Mozambique	2019	Part of a country (Northen Mozambique)	Displaced people and local residents affected by Cyclone Kenneth	Cyclone Kenneth, a category 3 storm, caused extensive destruction in northern Mozambique, it damaged water and sanitation infrastructure	Cyclone Kenneth destroyed sanitation infrastructure exacerbating re-existing vulnerabilities, leaving communities vulnerable to waterborne diseases, especially cholera	Cholera, Communicable diseases, acute respiratory illness (ARI)/influenza-like illness (ILI)
[33]	Containing community and communicable infectious diseases	India	From January 14 to March 3, 2019	Kumbh Mela in Prayagraj (pilgrimage and festival in Hinduism)	The millions of pilgrims attending the Kumbh Mela, including both semi-permanent residents and floating pilgrims	Mass gathering events—Large religious mass gathering during the Prayagraj Kumbh Mela, which posed significant public health risks due to the large influx of people, increasing the potential for disease transmission	Mass gathering events strained WASH, increasing the risk of communicable disease transmission, particularly acute respiratory illnesses, fevers, and skin infections, while also posing significant public health risks and threatening global health security due to the potential for rapid pathogen spread	Communicable diseases, including acute respiratory illness/influenza-like illness, acute fever, skin infections, acute diarrheal diseases, vector-borne diseases, vaccine-preventable diseases, and thermal events
[34]	Being prepared to manage infectious diseases even in crisis areas	Thailand	2010–2014 (with a focus on the 2013 vaccination campaign)	Refugee camp	Refugees in the Maela camp (43,645 individuals, primarily Burmese refugees)	Refugee crisis (overcrowding and poor sanitation in the Maela refugee camp)	Inadequate WASH conditions and dense populations in refugee camps necessitated vaccination as a critical addition to existing efforts under challenging logistical conditions	Cholera
[35]	Facing the global COVID-19 pandemic and internal issues	Philippines	2022	Bantayan Archipelago, part of Cebu Province in the Philippines, consisting of several islands and islets	Island populations	Weather disasters Typhoon Rai, known in the Philippines as Super Typhoon Odette	Extreme weather events and rising sea levels have delayed medical rescues and increased the vulnerability of island communities to emergencies, including COVID-19 and tuberculosis, highlighting the need for improved point-of-care testing (POCT) and diagnostics closer to remote communities	COVID-19, Tuberculosis, Dengue
[36]	Facing the global COVID-19 pandemic and internal issues	Nigeria	2020	IDPs camps in Abuja, Nigeria	Fefugee camp people (Victims of conflict—IDP camp residents)	Non-state armed groups (Insecurity due to conflicts, including Boko Haram insurgency and farmer-herder conflicts)	Displacement and insecurity have increased vulnerability to COVID-19 and hindered effective delivery of health promotion messages and vaccines	COVID-19
[37]	Being prepared to manage infectious diseases even in crisis areas	Yemen	Not specified, refers to the operational period of the surveillance systems (2014–2016)	Nationwide, health facilities	General population at risk (two-thirds of the population)	Ongoing civil war and humanitarian crisis impacting the healthcare system—Yemen is in crisis by 2014/2015	The degradation of healthcare infrastructure during the humanitarian crisis has led to a high incidence of malaria, with an increased risk of outbreaks, significant morbidity, and mortality, underscoring the need for improved surveillance systems	Malaria
[38]	Spread of paediatric infectious diseases	Nigeria	2017–2018	IDPs camps and Local Government Areas in Borno State, Nigeria	Children aged 9–59 months, especially those in IDP camps and security-compromised areas	Ongoing insurgency by Boko Haram leading to significant security challenges in the region	The insurgency disrupted routine immunisations, leading to large cohorts of unvaccinated children and increasing the risk of measles outbreaks, with security challenges making some areas completely inaccessible for vaccination campaigns	Measles
[28]	Being prepared to manage infectious diseases even in crisis areas	Uganda	2016–2020 (Plan development and implementation)	Nationwide	General population, including refugees	Multiple hazards including natural disasters, disease outbreaks, and technological and man-made disasters (e.g., terrorist attacks, floods, epidemics)	Uganda’s ecological vulnerability and public health emergencies have exacerbated the burden of communicable diseases, necessitating a coordinated, multi-sectoral response to prevent, detect, and respond to public health threats, while addressing gaps in preparedness	Cholera, Malaria, Typhoid fever, Meningitis, Viral Hemorrhagic Fevers (VHF), Hepatitis E, Avian influenza, Measles, Rift Valley Fever (RVF), Plague, Zika
[29]	Containing community and communicable infectious diseases	Uganda	2018–2019	National level, including high-risk districts bordering the DRC	General population, health workers	Various public health emergencies, including disease outbreaks and natural disasters, and refugee influx from DRC due to civil unrest and conflict	The high risk of EVD transmission due to cross-border movement, trade, and refugee influx from DRC prompted intensive preparedness activities, including community engagement, surveillance, vaccination, and the establishment of isolation units	Ebola Virus Disease (EVD), Viral Haemorrhagic Fever (VHF)
[39]	Spread of paediatric infectious diseases	South Sudan	2017	2 Outpatient Therapeutic Program centres—Bentiu Protection of Civilian site in Rubkona County, Unity State, South Sudan	Internally displaced children under five years old receiving nutrition services	Protracted war in South Sudan leading to severe humanitarian crises, including high levels of malnutrition and disrupted health systems	The protracted war has particularly affected children, leading to malnutrition, lack of vaccinations, and greater complications from infectious diseases such as measles, with ongoing conflict and displacement limiting access to health services, including routine immunisations	Paediatric vaccine-preventable diseases (e.g., measles, polio)

**Article**	**Intervention**						**Conclusion**	**JBI critical appraisal **[27]** overall score (%)**
	***Intervention***	***Materials***	***Authority***	***Result and Intervention Efficacy***	***Advantages***	***Disadvantage/Difficult to implementation***		
[31]	Organisational intervention with multipronged approach (e.g. treatment centres, surveillance, WASH, vaccines): Expansion of epidemiologic and laboratory surveillance, cholera treatment centres, beds, oral rehydration points, local investigations, WASH services, risk communication, community engagement, and Oral Cholera Vaccination (OCV) campaigns	Cholera treatment centres, oral rehydration points, OCVs, WASH services	Centres for Disease Control and Prevention (CDC), Ministère de la Santé Publique et de la Population, Pan American Health Organization, Médecins Sans Frontières	Over 20,000 suspected cholera cases were reported, leading to improved surveillance and response strategies. Although case counts have declined, transmission persists, necessitating ongoing efforts. The approach effectively reduced morbidity and mortality, with enhanced surveillance and targeted vaccination lowering the cholera fatality rate to below 1%. However, continued vigilance is essential, particularly in high-transmission areas, to maintain control	Utilization of existing technical capacity and experience from the previous cholera response; multipronged approach helps address complex challenges	Infrastructure deficiencies, insecurity and social unrest complicate response operations, scarcity of safe and treated water, delays in death reporting. High operational costs, need for multisectoral interventions and continuous infrastructure improvements	A multisectoral response is essential to control the cholera outbreak in a complex humanitarian crisis setting, need to improve surveillance, case management, access to WASH services, and targeted vaccination campaigns	87,5
[32]	Organisational intervention with multipronged approach (e.g. treatment centres, surveillance, WASH, vaccines): Social mobilization campaigns, establishment of cholera treatment centres, improvement of WASH infrastructure, and vaccination campaigns. OCV was administered, with over 252,448 people immunized	OCV, cholera kits, water sanitizers, hygiene kits, treatment centres, WASH interventions (e.g., well disinfection, water trucking)	Mozambique's Ministry of Health, WHO, Médecins Sans Frontières, OCHA, CDC, UNICEF, and various local and international partners	The cholera outbreak was efficiently controlled, with no fatalities reported by 31 May 2019. Over 252,000 people were vaccinated, leading to a significant decline in new cases after 5 May 2019. Effective coordination and prior experience in managing cholera outbreaks were crucial in mitigate the disease's spread	The multimodal approach, with the involvement of supra-governmental structures that can help local governments to give faster and more effective responses, is functional, as demonstrated by the lower number of events and deaths in the same Region following this meteorological event compared to in the past (see epidemic after a cyclone in 2015)	The intervention faced financial constraints, and the need for sustained funding was highlighted. The devastation of infrastructure complicated the logistics of response efforts	If future calamites like cyclones Idai or Kenneth occur, there shall be at least the same level of commitment between the government, NGOs and the population to control cholera and other diarrheal diseases as it happened this year. Yet, it is important to be realistic and prepare strategies to accommodate scenarios in which there is insufficient financial assistance, as it initially occurred during cyclone Idai. It would be a good idea for the government to maintain a contingency fund enough for such kind of emergencies	100
[33]	Surveillance intervention with multipronged approach (e.g. water quality monitoring, vector control, communicable diseases surveillance): A comprehensive onsite disease surveillance system was implemented, including indicator-based surveillance and event-based surveillance for 22 acute diseases/syndromes. Health facilities reported data daily, and an incident command centre was established for real-time analysis and response. The intervention also included water quality monitoring, vector control, and health education campaigns	Surveillance system with indicator-based surveillance and event-based surveillance, rapid diagnostic test kits, web-based reporting platform, and public health infrastructure for monitoring and response	State (provincial government of Uttar Pradesh) in coordination with the National Centre for Disease Control, WHO, CDC, and other stakeholders	The surveillance system identified 156,154 reportable conditions, 95% of which were communicable diseases, including acute respiratory illness (35%), acute fever (28%), and skin infections (18%). It successfully detected and managed two outbreaks (acute gastroenteritis and chickenpox), generating 12 early warning signals. The rapid response to these signals effectively controlled the outbreaks, preventing any major public health emergencies during the event	The intervention established a robust public health surveillance system that allowed for early detection and management of potential outbreaks. It also provided valuable data for future mass gatherings and improved the capacity of the local health system	Challenges included initial data entry errors due to unfamiliarity with the new system, and limited laboratory capacity for confirming outbreaks, which required presumptive case management	The study highlighted the importance of disease surveillance in managing public health risks during mass gatherings. The successful implementation of the surveillance system at the Prayagraj Kumbh Mela provided a model for future events, emphasizing the need for investment in public health planning, epidemic intelligence, and enhanced laboratory capacity	100
[34]	Modelling and organising an intervention (e.g. evaluating strategies): Theoretical model (SIWR-based transmission model) applied to a real-world setting to planning vaccination campaign. A cholera vaccination campaign using the Shanchol OCV, administered to 81% of the camp population (64% received both doses), informed and evaluated by a theoretical transmission model	Mathematical model—SIWR-based transmission model (theoretical model to evaluate and guide vaccination strategies); Shanchol OCV	NGOs, Première Urgence Aide Médicale Internationale, supported by the CDC and Thailand's Ministry of Public Health	Pre-vaccination, even with less than full coverage, significantly reduced cholera cases, and reactive vaccination proved effective, though timing was critical. Following the vaccination campaign, no cholera cases were reported in 2013 or 2014, demonstrating the campaign's success in preventing outbreaks	The vaccination campaign was effective even with less than full population coverage; the theoretical model provided valuable insights to optimise the intervention strategy	Potential logistical challenges in administering two doses of the vaccine, particularly in reactive vaccination scenarios where time is critical. Additionally, the mathematical model might not give precise data for variables such as hyper infectiousness, asymptomatic infections, or errors in disease reporting, which could lead to an overestimate of the total number of cases	Vaccination, combined with ongoing WaSH efforts, is an effective strategy to control cholera in refugee camps. They emphasise the importance of quick vaccine distribution in reactive scenarios, and demonstrate the utility of mathematical modelling in guiding public health interventions	88,9
[35]	Organisational intervention with multipronged approach (e.g. mapping, POCT): Geographic Contour Mapping: Analysis of sea and land ambulance rescue times to identify areas with prolonged rescue times. POCT Strategies: Design and implementation of POCT strategies for critical conditions like acute myocardial infarction, infectious diseases, and emergencies exacerbated by climate change. Spatial Care Paths: Development of the fastest routes for emergency care, integrating POCT to enhance decision-making and improve outcomes	POCT devices (e.g., glucose meters, pulse oximeters), ambulances equipped with basic diagnostic tools, and mobile testing units for COVID-19 and other diseases	Local healthcare facilities, emergency medical services, and public health teams in the Bantayan Archipelago, with research support from Cebu Technological University	Positioning POCT closer to vulnerable and remote populations can reduce health disparities, especially during extreme weather events. The study highlighted ambulance delays and stressed the need for strategic POCT placement. Integrating geographic analysis into healthcare planning and implementing spatial care paths is expected to improve healthcare delivery and outcomes during emergencies in remote areas	Improved access to diagnostics and emergency care. Enhanced community resilience against extreme weather events and health crises	High cost and complexity of implementing advanced POCT and upgrading local healthcare facilities. Potential logistical challenges in maintaining and operating POCT equipment in remote and harsh environments	POCT should be positioned upstream close to homes and island populations that have prolonged rescue time contours. Geospatially optimized point-of-need diagnostics and distributed prehospital testing have high potential to improve health outcomes	71,4
[36]	Communication intervention (e.g. Counselling, visual illustration): Visual illustrations on COVID-19 vaccination were shown to participants alongside counselling sessions to encourage vaccine acceptance	Visual illustrations and counselling	Department of Mass Communication	Visual illustrations and counselling effectively improved attitudes, self-efficacy, task efficacy, positive outcome expectancy, and intentions towards COVID-19 vaccination among victims of insecurity	Effective in increasing vaccine acceptance among a vulnerable population; applicable to similar settings. Easy to reproduce	Focused only on IDP camp residents; did not include victims outside the camps or those affected by natural disasters. The effect of visual illustrations and counselling isn't analysed separately	The study highlights the importance of tailored health communication interventions in promoting vaccine acceptance among vulnerable groups in conflict settings	100
[37]	Surveillance intervention: Evaluation and comparison of two malaria surveillance systems: the Integrated Malaria Surveillance System (IMSS) and the Early Disease Electronic Warning System (eDEWS)	Data collection tools, malaria registration books, eDEWS reporting forms, rapid diagnostic tests, and computers for data entry and analysis	National Malaria Control Program (NMCP), Ministry of Public Health and Population, supported by the WHO	The IMSS was useful for assessing malaria burden but had poor overall performance and was not effective for outbreak detection. In contrast, eDEWS performed well, particularly in outbreak detection, though it was limited to this function. To maximise their strengths, both systems need to be integrated	IMSS: Useful for long-term planning and monitoring of malaria trends eDEWS: Excellent data quality and timeliness, effective in early outbreak detection, and good overall performance. Integration of both systems would leverage the strengths of each, improving malaria surveillance and outbreak response.—Integration recommended to maintain advantages of both systems and improve sustainability	IMSS: Poor performance in outbreak detection, limited flexibility, low representativeness, and instability due to reliance on external funding eDEWS: While performing well in outbreak detection, it does not fully address all NMCP needs and could suffer from sustainability issues if funding is cut	Both systems should be integrated to combine the advantages of eDEWS (simplicity, data quality, outbreak detection) and IMSS (data for burden assessment and future planning). This integration could ensure a sustainable and comprehensive malaria surveillance system in Yemen	100
[38]	Organisational intervention (e.g. strategies to improve vaccinations): The measles vaccination campaign involved strategies like fixed and temporary posts, Reaching Inaccessible Children (RIC), Reaching Every Settlement (RES), and combined human-animal vaccination teams targeting nomadic populations	Vaccines (Measles vaccine), temporary posts (RIS and RES)	Government of Nigeria, supported by partners such as WHO and UNICEF	The campaign vaccinated 1,660,889 children, with 20% receiving their first dose, and achieved a 72% coverage rate according to the post-campaign survey, though this was the lowest in Nigeria. Insecurity in certain regions prevented full implementation, leading to incomplete coverage and a significant increase in measles cases, particularly in security-compromised areas. Reported measles cases rose from 270 in 2017 to 757 in 2018, highlighting the impact of these challenges on outbreak control	Utilised various strategies to reach different populations, such as combining human and animal vaccination teams for nomadic groups, and leveraging military support for access to insecure areas	Security challenges limited access to some areas, and certain strategies could not be fully implemented. Additionally, the discrepancy between administrative and survey-reported coverage indicates issues with data management	Need for a standalone campaign in conflict-affected states, continuous engagement with the military for improved security coordination, and the reestablishment of vaccination posts at IDP camp entry points. Regular use of the WHO measles risk assessment tool for better-targeted interventions	100
[28]	Modelling and organising an intervention with multipronged approach (e.g. risk profiling, risk analysis): Development of the National Multi-hazard Emergency Preparedness and Response Plan (NMEPRP) using a logic model to harmonize processes and guide stakeholders; risk profiling using the Strategic Tool for Analysis of Risks (STAR); coordination of preparedness and response across multiple sectors	Strategic Tool for Analysis of Risks (STAR), national guidelines, Standard Operating Procedures (SOPs), risk communication strategies, laboratory networks	Uganda Ministry of Health, supported by the Office of the Prime Minister and other stakeholders, WHO, CDC, other development partners and non-governmental organisations	The logic model and STAR tool successfully guided the development of Uganda's comprehensive national emergency preparedness plan. This process involved identifying key public health preparedness and response capabilities, categorizing risks, and defining necessary preparedness actions. The country's operational response capacity for high-risk public health events was significantly enhanced, improving its ability to address public health threats	Comprehensive multi-sectoral approach; inclusion of diverse stakeholders; alignment with WHO's Strategic Framework for Emergency Preparedness; use of proven risk assessment tools; establishment of clear roles and responsibilities for emergency response. Applicable nationally with involvement of various sectors and partners	Requires ongoing funding and resources for implementation; complexity in coordinating across multiple sectors and levels of government; need for continuous updating and training	The multi-hazard plan is ready for implementation and will contribute to strengthening emergency preparedness and resilience in Uganda	85,7
[29]	Organisational intervention with multipronged approach (e.g. treatment centers, surveillance, training, vaccines): Activation of the Public Health Emergency Operations Centre, National Task Force, and District Task Forces; establishment of an Incident Management Team; risk classification of districts; surveillance; training of health workers; vaccination campaign for frontline workers; construction of Ebola Treatment Units; and cross-border collaboration	Ebola Treatment Units, Personal Protective Equipment, rVSV-ZEBOV vaccine, risk communication materials, surveillance tools, WHO EVD preparedness checklist	Uganda Ministry of Health, supported by WHO and other international partners	Significant progress was made in EVD preparedness in Uganda, with no confirmed cases during the study period. The country achieved 92% readiness, successfully vaccinated 4,419 frontline health workers, and established effective surveillance and rapid response mechanisms. However, ongoing vigilance and sustained efforts are necessary due to the prolonged outbreak	Quick activation and coordination; multi-sectoral and multi-disciplinary approach; effective use of past experiences and existing structures; successful vaccination and training programs	Ongoing risk of cross-border transmission due to the prolonged outbreak in DRC; resource fatigue; challenges in maintaining preparedness momentum and funding; strain on border screening efforts	The rapid activation of preparedness mechanisms and multi-sectoral collaboration were key to Uganda's success in preventing EVD importation; sustained efforts and resources are required to maintain preparedness and respond to future outbreaks	100
[39]	Organisational intervention (e.g. integration of services to improve vaccination coverage): Immunization services were integrated into existing nutrition services at Outpatient Therapeutic Program centres. This included on-site vaccinations during nutrition visits, tracking of children who missed vaccinations, and providing health education on immunizations during community outreaches. The primary outcome measure was receipt of appropriate antigens by children assessing nutrition services and the secondary outcome measure was dropout in vaccination	Integration of two existent services [Ready-to-use therapeutic food, vaccines (e.g., oral polio vaccine, pentavalent vaccine, measles vaccine), immunization registers, child health cards, and Mid-upper arm circumference tapes for malnutrition screening]	UNICEF, in collaboration with local health authorities and nutrition partners	The integration of immunization into nutrition programmes significantly increased vaccine coverage, with a notable rise in the number of children immunized. The study found that children in Outpatient Therapeutic Program centres had a lower likelihood of missing vaccinations compared to those in Primary Health Care Centres, resulting in reduced dropout rates for vaccines	Improved vaccine coverage and reduced missed opportunities for vaccinations among vulnerable populations. It demonstrated the feasibility and effectiveness of integrating services in resource-constrained settings	The intervention relied heavily on incentives (e.g., Ready-to-use therapeutic food) to encourage participation, which might not be sustainable long-term. Also, integrating services requires careful planning to avoid overburdening existing systems	The integration of immunization services into nutrition programmes effectively increased vaccination coverage among internally displaced children in South Sudan. The approach is in line with global strategies for child health and should be considered for broader implementation	100

### Study characteristics

The 11 selected studies encompass various periods, spanning from 2010 to 2023, and covered various countries, Haiti [31], India [33], Mozambique [32], Nigeria [36, 38], the Philippines [35], South Sudan [39], Thailand [34], Uganda [28, 29], and Yemen [37]. They analysed conflicts [29, 31, 36–39], mass gathering events [33], natural disasters [28, 29, 32, 35], and refugee crises [28, 29, 34] affecting various populations, including children [31, 39], the general population [28, 29, 36, 37], island populations [35], pilgrims [33], and refugees [28, 29, 32, 34, 36]. The infectious diseases addressed included cholera [29, 31, 32, 34], COVID-19 [35, 36], Ebola [29], malaria [28, 37], measles [28, 38, 39], tuberculosis, dengue [35], other communicable diseases [28, 32, 33], and other paediatric vaccine-preventable diseases [39]. The interventions employed varied, encompassing communication strategies [36], modelling and organisational efforts [28, 34], organisational interventions [29, 31, 32, 35, 38, 39], and surveillance initiatives [33, 37]. Some interventions require a multipronged approach [28, 29, 31–33, 35].

To enhance the accessibility of the results, the articles have been categorised based on the type of infectious disease, setting and intervention implemented; this categorisation did not influence the data synthesis methodology. The categories are as follows: containing community and communicable infectious diseases [29, 31–33], being prepared to manage infectious diseases even in crisis areas [28, 34, 37], facing the global COVID-19 pandemic and internal challenges [35, 36], and spreading paediatric infectious diseases [38, 39].

Further details are provided in the Table 2.

#### Spread of paediatric infectious diseases

A study [39], conducted in outpatient therapeutic programmes (OTP) centres in South Sudan between January and December 2017, focused on children under age 5. The prolonged war led to malnutrition, a lack of vaccinations, and increased complications from diseases such as measles. Compared with primary health care centres, integrated immunisation and nutrition services at OTP centres during outreaches significantly improved vaccination rates and reduced dropout rates.

In Nigeria [38], between January 2017 and December 2018, conflict led to population displacement, limiting access to healthcare and safe water, and causing measles and other vaccine-preventable disease outbreaks among children aged 9–59 months. The Reaching Every Settlement (RES) strategy aimed to vaccinate 7000 children, and 4622 (68%) received the measles vaccine, resulting in 72% state-wide coverage. However, measles incidence has increased from 22.7 to 101.8 per million. Despite efforts, children in inaccessible areas remain unvaccinated, highlighting the need for strategies to reach these populations.

#### Facing the global COVID-19 pandemic and internal issues

In the Bantayan Archipelago, Philippines, Super Typhoon Odette in late 2021 and 2022 worsened material shortages and delayed rescues, impacting patient care and complicating the spread of COVID-19, tuberculosis, and other infectious diseases [35]. The government's response included needs assessments, facility inspections, and ambulance rescue time data collection. Researchers have mapped and compared rescue routes, developing spatial care paths. Point-of-care testing near homes and island populations with prolonged rescue times, using geospatially optimised distributions, can save lives by ensuring timely diagnostics and care, significantly improving health outcomes. Integrating these diagnostics into public health strategies enhances geographic health resilience, especially for isolated populations.

In Nigeria, nonstate armed groups disrupted security, law, and order, impacting healthcare delivery, especially during the pandemic in 2021 [36]. This exacerbated the situation for internally displaced persons (IDPs) in camps. The Department of Mass Communication implemented an intervention using visual illustrations to highlight the importance of COVID-19 vaccination, which increased adherence among the 470 conflict victims who completed the questionnaire. The intervention's main advantage is its potential applicability in various settings. However, it is unclear whether the increased adherence was due to visual communication or broader educational efforts.

#### Being prepared to manage infectious diseases even in crisis areas

In Thailand, a mathematical model was used to evaluate the evolution of a cholera outbreak and the impact of different vaccination scenarios under logistical constraints in a refugee camp from 2010 to 2014 [34]. The camp, with a dense population and inadequate water, sanitation, and hygiene (WASH) conditions, saw no cases during those years because of vaccination efforts. This suggests that vaccination can be effective even in challenging logistical contexts, highlighting the potential benefits of administering one dose to more people rather than two doses to fewer people in reactive vaccination scenarios or as a preventive measure in refugee camps, even without ongoing outbreaks.

Yemen has seen its situation worsen due to escalating internal conflicts [40]. The National Malaria Control Program (NMCP) evaluated two malaria surveillance systems, the Integrated Malaria Surveillance System (IMSS) and the Early Disease Electronic Warning System (eDEWS), from 2009 to 2016 [37]. The IMSS was useful for assessing malaria burden but had poor overall performance and was ineffective for outbreak detection. Conversely, eDEWS excelled in outbreak detection but was limited to that function. To enhance malaria surveillance and outbreak response, integrating both systems is recommended to combine their strengths.

In Uganda, a national multi-hazard emergency preparedness and response plan was developed using a preparedness logic model to address public health emergencies and multiple hazards, including disease outbreaks (e.g., cholera, malaria, typhoid, meningitis, hepatitis E, measles, influenza, Zika, and plague) and natural disasters, with a focus on refugee camps [28]. The plan involved the Ministry of Health of Uganda, the World Health Organisation (WHO), the centres for Disease Control and Prevention (CDC), and other development partners and nongovernmental organisations (NGOs) in planning and implementing response strategies, identifying risks, and developing operational capacities. This plan has significantly strengthened Uganda's emergency preparedness and resilience, ensuring timely and adequate responses to emergencies.

#### Containing community and communicable infectious diseases

Haiti was impacted by gang violence, population displacement, social unrest, and insecurity, which destroyed public health infrastructure and sanitation services, leading to an increase in cholera cases. Involving the CDC and other organisations, the intervention, from September 2022 to January 2023, focused on children under 10 years old [31] and included epidemiologic surveillance, case management, rehydration points, WASH service improvements, oral cholera vaccination (OCV), and community engagement. These efforts have reduced morbidity and mortality, improved surveillance, and led to targeted vaccination campaigns. However, infrastructure deficiencies, insecurity, scarcity of safe water, delays in reporting, high operational costs, and multisectoral interventions pose significant challenges.

Mozambique was severely impacted by Cyclone Kenneth in 2019, worsening the spread of cholera and disrupting disease containment plans [32]. An intervention led by the Ministry of Health with support from the WHO and other organisations involved social mobilisation, hygiene promotion, cholera treatment centres, an OCV campaign, and WASH interventions. This multimodal approach reduced the number of cholera cases, with no fatalities reported. While external financial support enabled a swift response, establishing a national emergency fund for future crises is essential.

From January to March 2019, mass gatherings in Indian cities increased health risks from communicable diseases and strained health systems [33]. A health coordination committee conducted an all-hazard risk assessment and reviewed the Integrated Disease Surveillance Programme, enhancing weekly passive surveillance with daily onsite monitoring. This system identified diseases with epidemic potential and severity. The implementation of epidemic intelligence-enabled surveillance effectively addressed public health threats, particularly acute respiratory illnesses and influenza-like illnesses, and improved data for healthcare workforce deployment and planning for drug and vaccine supplies. However, data entry errors were frequent. Inadequate residual chlorine in 20% of the water samples triggered early warnings for acute diarrhoeal diseases, vector-borne diseases, and vaccine-preventable diseases, with two outbreaks quickly controlled.

From 2018 to 2019, Uganda faced public health emergencies, including natural disasters and refugee crises [29]. Led by the Uganda Ministry of Health, the WHO, and partner organisations, the intervention involved activating coordination mechanisms, training health workers, risk communication, and simulation exercises, particularly in high-risk districts and border points, owing to the threat of Ebola virus disease (EVD) from cross-border movement and refugee influx from the Democratic Republic of the Congo (DRC). Preventive strategies, including community engagement, surveillance, ebola vaccination (rVSV-ZEBOV), and isolation units, were implemented and effectively prevented the EVD spread, proving cost-effective, with no cases during the study period.

## Discussion

The review spans different geographical regions and crises, highlighting the impacts of armed conflict, natural disasters, and public health emergencies. The findings underscore the critical need for coordinated, multisectoral interventions to address the spread of infectious diseases and improve health outcomes during humanitarian crises. Effective strategies identified include the integration of services, the implementation of epidemic intelligence-enabled surveillance systems, the optimisation of vaccination campaigns in challenging logistical contexts and the improvement of WASH services and point-on-care. The interventions were supported by international agencies and NGOs, highlighting the importance of international collaboration. These interventions, despite varying in cost and complexity, share a common goal: to increase resilience and preparedness in crisis-affected areas.

The link between conflict and infectious outbreaks is well documented, and the WHO supports the development of flexible and sustainable interventions to respond to changing conditions [41]. In the case of outbreaks of vaccine-preventable diseases, countries should reevaluate their vaccination policies to ensure high coverage [42], particularly if rates are below WHO recommendations. Meningitis outbreaks, measles transmission and epidemics in disaster contexts, such as the one following the 2005 earthquake in Pakistan [43], demonstrate that diseases often originate from pathogens already present in the population that find favourable conditions to cause larger epidemics [44]. Vaccination remains a key preventive measure, and there are several examples in the literature and in recent history. During the migration crisis from the DRC, Uganda used rVSV-ZEBOV to reduce the risk of the spread of Ebola [29]. Vaccination campaigns for typhoid, such as after an earthquake in Nepal in 2015 [45] or a cyclone in India in 2004 [46], have proven effective. Integrating vaccination efforts with WASH awareness can create a robust disease control strategy in disaster-affected regions [46]. In Haiti [31], as well as in Mozambique aftermath of Cyclone Kenneth [32], multimodal strategies combining OCV and other interventions led to a reduction in morbidity and mortality. The use of optimised immunisation strategies, which prioritise the vaccination of larger population groups with fewer doses rather than smaller groups with more doses, can be efficient in optimising resources to reduce the spread of infectious diseases, as experienced in Thailand [34]. Vaccinations are extremely important to children, and children are highly vulnerable during humanitarian crises because of the loss of health, nutrition, hygiene, and security [41]. A study conducted in Somalia demonstrated that conflict exacerbates the indirect costs of child mortality related to measles [47]. In South Sudan [39], a coordinated strategy involving the integration of immunisation and nutrition services into OTP significantly increased immunisation rates and reduced dropout rates while being cost-effective. In Nigeria [28], the RES strategy has been helpful in increasing vaccination coverage, but the incidence of measles has remained high because of challenges in reaching the entire population. These cases underscore the difficulty of achieving high vaccination coverage in unstable, conflict-affected settings. Multimodal strategies, including the use of geographic information systems, are effective in increasing vaccination coverage [48]. Expanding vaccination coverage in conflict zones is essential to prevent future pandemics because a measles outbreak could have devastating consequences in conflict-affected areas [49]. In response to the recent conflict affecting the population of Gaza [50], the WHO launched a polio vaccination campaign targeting the region. The campaign has already achieved significant results, particularly in central Gaza, underscoring the critical role of immunisation in managing public health risks during humanitarian crises [51].

Moreover, the pandemic experience deserves particular focus. The global health crisis caused by COVID-19 has posed significant challenges, particularly for LMICs, which face severe economic and healthcare resource constraints. These countries struggled with the lack of personal protective equipment and the inadequacy of nonpharmaceutical interventions, such as lockdowns and school closures, which had profound socioeconomic repercussions not suitable for LMICs [52, 53], underscoring the importance of context-specific interventions considering health infrastructure and socioeconomic factors [54]. After Super Typhoon Odette, in the Philippines, the government's response to the spread of COVID-19, tuberculosis, and other infectious diseases [35] included needs assessments, facility inspections, rescue time and point-of-care testing near homes and island populations, the use of geospatially optimised distributions, and saving lives by ensuring timely diagnostics and care. In crisis setting, as with IDPs camps [36], a visual communication strategy can be cost-effective to increase adherence to vaccination (e.g. COVID-19 vaccination). However, the scarcity of economic resources and healthcare products, such as medicines, vaccines, diagnostics, and devices, further complicated the diagnosis and treatment of COVID-19 and other diseases, especially in areas with limited access to essential services such as WASH [55, 56].

Given the importance of prevention, the essential role of surveillance must also be taken into account. To prevent public health emergencies, it is essential to understand risk factors and maintain continuous surveillance with timely reporting for early outbreak detection and rapid response. Robust surveillance systems are critical for timely detection and rapid response to disease outbreaks [31, 33]. During conflict and cholera outbreaks [31], surveillance strategy, which identified hotspots and efficiently directed resources, can be effective. Similarly, India’s epidemic intelligence-enabled surveillance during mass gatherings successfully addressed public health threats and facilitated rapid responses [33]. In Yemen, the integration of surveillance systems, such as IMSS and eDEWS, enables more effective responses by leveraging the strengths of each system [37]. Predictive models are valuable for resource-limited LMICs, as they help predict disease spread and guide public health decisions [57, 58].

Furthermore, prevention also relies on environmental and healthcare containment strategies. WASH service improvements are fundamental to reducing waterborne disease transmission [59]. Providing clean water, building sanitation facilities, and implementing hygiene education programs significantly impact public health. In Mozambique and Uganda, WASH improvements have led to a decline in diarrhoeal diseases [32, 60]. However, the literature highlights gaps in the impact of WASH interventions on health outcomes [61]. Sustainability and long-term strategies are necessary for sustained preparedness and outbreak prevention efforts [62]. The rise of cholera cases in Haiti [31], affected by gang violence, population displacement, social unrest, and insecurity, and in Mozambique, impacted by Cyclone Kenneth [32], was managed, among other treatments, through rehydration points, WASH service improvements, and hygiene promotion, reducing morbidity and mortality. In these situations, external financial support has been pivotal in strengthening healthcare systems and implementing disease control measures. For those reasons, governments should develop strategies to manage scenarios with limited financial assistance, including maintaining contingency funds for emergencies [63].

Prevention is not always effective, for this reason, it is essential to be prepared in advance. After disasters, migration, water and foodborne diseases, and compromised personal hygiene [64] can lead to diarrhoeal diseases becoming a cause of death [65]. After the Bam earthquake in Iran, 1.6% of the 75,586 people were affected by diarrhoeal disease due to poor hygiene and overcrowding [66]. In the 2001 El Salvador earthquake, 22% of examined individuals had gastrointestinal infections, and 30% had respiratory infections [67]. Overcrowding, poor ventilation, and destruction of healthcare infrastructure exacerbate acute respiratory infections, with additional risks from endemic diseases and low vaccination coverage [4]. In Guangzhou, China, climatic changes have increased mosquito populations and dengue transmission [68]; furthermore, arboviruses are emerging as a major global concern, highlighting the need for ongoing surveillance and improved healthcare preparedness [69]. To be ready for this type of event, in Uganda, a national multihazard emergency preparedness and response plan was implemented to improve preparedness, build resilience, and ensure timely and adequate responses to emergencies [28]. Effective disaster management and resource planning can prevent the spread of infectious diseases and control emerging threats [70]. However, this often requires collaboration between governmental and NGOs, particularly in countries with limited institutional capacity, allowing for pooling resources and sharing decision-making responsibilities [71]. Nonprofit organisations, the private sector, volunteer groups, and communities have unique skills, contributing at various levels to achieving the objectives of the complex disaster management process [72], highlighting the importance of collaboration among stakeholders at the local, national, and international levels [73], as well as cross-border collaborations [74]. Coordinated efforts among countries, such as those against Ebola outbreaks in West Africa [34], enhance surveillance, streamline response strategies, and facilitate resource sharing.

Moreover, the scientific literature provides considerable evidence on the importance of being prepared for the containment of infectious diseases. Containing communicable infectious diseases in LMICs is challenging because factors such as overcrowded living conditions and the scarcity of essential resources [61], inadequate infrastructure, and financial constraints [75]. A comprehensive approach to disease prevention requires coordinated efforts. The transmission of infections to neighboring countries has been linked to ill-prepared health systems and poor intergovernmental coordination, leading to inadequate disease surveillance, insufficient infection prevention and control, and poor clinical care [34, 74]. When risks are known in advance, such as during large mass gatherings, authorities must plan ahead for water, sanitation, hygiene, and medical care infrastructure [33, 76]. The scalability and applicability of these strategies to other regions with similar challenges are important, even though tailored approaches based on specific regional risks and resources are necessary. After Cyclone Kenneth in Mozambique, the value of national and regional preparedness plans became evident in reducing the spread of cholera due to an efficient response guided by Mozambique Humanitarian Response Plan [32]. In areas with limited healthcare infrastructure, training community health workers and using standardised treatment protocols have proven effective [77]. Similarly, Uganda implemented an emergency preparedness and response plan with WHO support and community engagement to address Ebola outbreaks [34, 78]. Patient management is essential for disease control, improving patient outcomes and reducing infection spread [79]. Risk communication is critical for informing and educating the public, ensuring timely information dissemination and public cooperation in interventions. In Mozambique, efforts to control cholera faced resistance, especially in the north, where conspiracy theories suggested that the government introduced cholera to harm the population [32]. Multisectoral interventions include efforts in epidemiologic surveillance, case management, vaccination campaigns, WASH service improvements, and cross-border collaborations [54]. An integrated approach that combines individual, social, and structural strategies ensures a more effective response [80]. However, a multisectoral approach is needed for managing complex and diverse infectious diseases in various crisis settings.

### Policy

This systematic review highlights the importance of flexible strategies adaptable to different settings. To prevent infectious diseases in LMICs during humanitarian crises, careful planning, adequate resources, and close collaboration among international organisations, NGOs, and local governments are essential, confirming and supporting efforts already underway.

### Strengths and limitations

This systematic review has several strengths and limitation. First, the varying quality and design of the included studies, along with differences in data availability, may affect the reliability of the conclusions. To address this, we ensured the use of robust quality appraisal tools (e.g., JBI checklists) and a systematic synthesis approach. However, limitations such as potential publication bias, heterogeneity in study methodologies, and incomplete data reporting might have introduced some degree of variability in the reliability of our findings. These factors were considered during data synthesis, and their potential impact is acknowledged as a limitation of this study. Second, the systematic review focused on studies that specifically discussed the containment of infectious diseases during humanitarian crises, which led to the exclusion of studies that individually addressed the impact of humanitarian crises on infectious diseases, interventions to contain infectious diseases in LMICs, or the challenges of healthcare interventions during humanitarian crises. Third, while the diversity of crises and settings covered enables broader generalisation, it also presents challenges in drawing firm conclusions about the effectiveness of specific interventions due to the variability in contexts. Fourth, by covering a wide range of geographical areas, this review offers a comprehensive examination of many LMICs.

## Conclusion

LMICs are particularly vulnerable to the risks of infectious diseases during crises caused by armed conflict, natural disasters, and public health emergencies. Various strategies and interventions have been implemented worldwide to address the spread of communicable diseases. These actions can vary in cost and complexity, ranging from communication campaigns to vaccination and from targeted strategies to multirisk and multipronged interventions. The integration of multisectoral approaches, such as integrated vaccination campaigns, surveillance systems, and improvements in WASH services, is needed for containing the spread of diseases and protecting vulnerable populations. Proactive planning, especially in fragile healthcare settings, and collaboration between international organisations, NGOs, and local governments are essential. Notably, the majority of LMICs have required support from international organisations (e.g., the WHO) and have benefited from the application of standardised plans and strategies, highlighting the importance of knowledge sharing and international collaboration. These findings are even more significant considering the increase in armed conflicts and natural disasters, as well as the growing risks posed by emerging infections (e.g., dengue, monkeypox, oropouche), which threaten global security, particularly in LMICs.

## Supplementary Information

Supplementary Material 1.

Supplementary Material 2.

## Data Availability

No datasets were generated or analysed during the current study.

## Abbreviations

ARI: Acute respiratory illness
CDC: Centres for Disease Control and Prevention
DRC: Democratic Republic of the Congo
eDEWS: Early Disease Electronic Warning System
EVD: Ebola virus disease
IDPs: Internally Displaced Persons
ILI: Influenza-like illness
IMSS: Integrated Malaria Surveillance System
JBI: Joanna Briggs Institute
LMICs: Low- and middle-income countries
NCDC: National Centre for Disease Control
NGOs: Nongovernmental organisations
NMCP: National Malaria Control Program
OCHA: Office for the Coordination of Humanitarian Affairs
OTP: Outpatient therapeutic programmes
POCT: Point-of-care testing
PRISMA: Preferred Reporting Items for Systematic Reviews and Meta-Analyses
RES: Reaching Every Settlement
rVSV-ZEBOV: Recombinant vesicular stomatitis virus-Zaire Ebola virus
SPIDER: Sample, Phenomenon of interest, Design, evaluation, Research type
UNICEF: United Nations International Children's Emergency Fund
WASH: Water, sanitation, and hygiene
WHO: World Health Organisation
