# Supplementary material for: Building evidences in Public Health Emergency Preparedness (“BePHEP” Project)—a systematic review
Source: Int J Equity Health. 2025 Feb 11;24:41. doi: 10.1186/s12939-025-02382-w (PMC11817627; doi:10.1186/s12939-025-02382-w)
Supplement: Supplementary file 1 — Supplementary Material 1. [file 12939_2025_2382_MOESM1_ESM.docx]

| **Criteria** | **Search terms used for papers’ identification** |
| --- | --- |
| **Geographical area of interest** | ""Developing Countr*" OR "LMIC*" OR "Afghanistan" OR "Korea*" OR "South Sudan" OR "Burkina Faso" OR "Liberia" OR "Sudan" OR "Burundi" OR "Madagascar" OR "Syria*" OR "Central Africa*" OR "Malawi" OR "Togo" OR "Chad" OR "Mali" OR "Uganda" OR "Congo*" OR "Mozambique" OR "Yemen*" OR "Eritrea" OR "Niger" OR "Ethiopia" OR "Gambia*" OR "Rwanda" OR "Sierra Leone" OR "Guinea-Bissau" OR "Somalia" OR "Angola" OR "Jordan" OR "Philippines" OR "Algeria" OR "India" OR "Samoa" OR "Bangladesh" OR "Iran*" OR "Sao Tome and Principe" OR "Benin" OR "Kenya" OR "Senegal" OR "Bhutan" OR "Kiribati" OR "Solomon Islands" OR "Bolivia" OR "Kyrgyz*" OR "Sri Lanka" OR "Cabo Verde" OR "Laos" OR "Tanzania" OR "Cambodia" OR "Lebanon" OR "Tajikistan" OR "Cameroon" OR "Lesotho" OR "Timor-Leste" OR "Comoros" OR "Mauritania" OR "Tunisia" OR "Congo*" OR "Micronesia*" OR "Ukraine" OR "Ivoire" OR "Mongolia" OR "Uzbekistan" OR "Djibouti" OR "Morocco" OR "Vanuatu" OR "Egypt*" OR "Myanmar" OR "Vietnam" OR "Eswatini" OR "Nepal" OR "Zambia" OR "Ghana" OR "Nicaragua" OR "Zimbabwe" OR "Guinea" OR "Nigeria" OR "Haiti" OR "Pakistan" OR "Honduras" OR "Papua New Guinea" OR "Albania" OR "Fiji" OR "North Macedonia" OR "Argentina" OR "Gabon" OR "Palau" OR "Armenia" OR "Georgia" OR "Paraguay" OR "Azerbaijan" OR "Grenada" OR "Peru" OR "Belarus" OR "Guatemala" OR "Russia*" OR "Belize" OR "Indonesia" OR "Serbia" OR "Bosnia and Herzegovina" OR "Iraq" OR "South Africa" OR "Botswana" OR "Jamaica" OR "St. Lucia" OR "Brazil" OR "Kazakhstan" OR "St. Vincent and the Grenadines" OR "Bulgaria" OR "Kosovo" OR "Suriname" OR "China" OR "Libya" OR "Thailand" OR "Colombia" OR "Malaysia" OR "Tonga" OR "Costa Rica" OR "Maldives" OR "Turkiye" OR "Cuba" OR "Marshall Islands" OR "Turkmenistan" OR "Dominica" OR "Mauritius" OR "Tuvalu" OR "Dominican Republic" OR "Mexico" OR "Gaza" OR "El Salvador" OR "Moldova" OR "Equatorial Guinea" OR "Montenegro" OR "Ecuador" OR "Namibia" |
| AND | |
| **Timeframe** | PUBYEAR > 2018 AND PUBYEAR < 2024 |
| AND | |
| **Sample** | "Migration*" OR "Migrant*" OR "Displacement*" OR "Armed Conflict*" OR "Global Warming" OR "Conflict*" OR "War*" OR "Refugee*" OR "Climate Change" |
| AND | |
| **Phenomenon of Interest** | "Epidemic*" OR "Communicable Disease*" OR "Vaccin*" OR "Vector*" OR "Virus*" OR "Waterborne OR Outbreak*" OR "PHEIC" OR "VPD*" OR "infectious disease*" |
| AND | |
| **Evaluation** | "Surveillance" OR "Sanitation" OR "Preparedness" OR "Prevention*" OR "Biocontainment" OR "Infection Control" OR "Epidemic Intelligence" OR "Response" OR "PHEP" OR "Public Health" OR "early warning*" OR "EWAR*" OR "vaccin*" |
|  | AND |
|  | “Plan*" OR "Polic*" OR "Resilience" OR "Sustainability" |
|  | AND |
|  | "Prevalence" OR "Incidence" OR "Mortality" OR "Morbidity" OR "death*" OR "illness" OR "infection rate*" OR "outcome*" |

**Supplementary Table 1**. Search terms used for papers’ identification.
