# Supplementary material for: Building evidences in Public Health Emergency Preparedness (“BePHEP” Project)—a systematic review
Source: Int J Equity Health. 2025 Feb 11;24:41. doi: 10.1186/s12939-025-02382-w (PMC11817627; doi:10.1186/s12939-025-02382-w)
Supplement: Supplementary file 2 — Supplementary Material 2. [file 12939_2025_2382_MOESM2_ESM.docx]

| **JBI critical appraisal checklist [1] for** | **Studies reporting prevalence data** | | | **Textual evidence: narrative** | | | | **Economic evaluations*** | | **Analytical cross sectional studies** | | **Quasi-experimental studies** | | | **Qualitative research** | | **Textual evidence: policy/consensus guidelines** | |
| --- | --- | --- | --- | --- | --- | --- | --- | --- | --- | --- | --- | --- | --- | --- | --- | --- | --- | --- |
| **Article** |  | **[2]** | **[3]** |  | **[4]** | **[5]** | **[6]** |  | **[7]** |  | **[8]** |  | **[9]** | **[10]** |  | **[11]** |  | **[12]** |
| Q1 | Was the sample frame appropriate to address the target population? | Yes | Yes | Is the generator of the narrative a credible or appropriate source? | Yes | Yes | Yes | Is there a well-defined question? | Yes | Were the criteria for inclusion in the sample clearly defined? | No | Is it clear in the study what is the 'cause' and what is the 'effect'? | Yes | Yes | Is there congruity between the stated philosophical perspective and the research methodology? | Yes | Are the developers of the policy/ consensus guideline (and any allegiences/affiliations) clearly identified? | Yes |
| Q2 | Were study participants sampled in an appropriate way? | Unclear | Yes | Is the relationship between the text and its context explained? (where, when, who with, how) | Yes | Yes | Yes | Is there comprehensive description of alternatives? | Yes | Were the study subjects and the setting described in detail? | Yes | Was there a control group? | Yes | Yes | Is there congruity between the research methodology and the research question or objectives? | Yes | Do the developers of the policy/ consensus guideline have standing in the field of expertise? | Yes |
| Q3 | Was the sample size adequate? | Yes | Yes | Does the narrative present the events using a logical sequence so the reader or listener can understand how it unfolds? | Yes | Yes | Yes | Are all important and relevant costs and outcomes for each alternative identified? | Yes | Was the exposure measured in a valid and reliable way? | Yes | Were participants included in any comparisons similar? | Yes | Yes | Is there congruity between the research methodology and the methods used to collect data? | Yes | Are appropriate stakeholders involved in developing the policy/guideline and do the conclusions drawn represent the views of their intended users? | Yes |
| Q4 | Were the study subjects and the setting described in detail? | Yes | Yes | Do you, as reader or listener of the narrative, arrive at similar conclusions to those drawn by the narrator? | Yes | Yes | Not Applicable | Has clinical effectiveness been established? | Yes | Were objective, standard criteria used for measurement of the condition? | Yes | Were the participants included in any comparisons receiving similar treatment/care, other than the exposure or intervention of interest? | Yes | Yes | Is there congruity between the research methodology and the representation and analysis of data? | Yes | Are biases due to competing interests acknowledged and responded to? | Yes |
| Q5 | Was the data analysis conducted with sufficient coverage of the identified sample? | Yes | Yes | Do the conclusions flow from the narrative account? | Yes | Yes | Yes | Are costs and outcomes measured accurately? | Yes | Were confounding factors identified? | No | Were there multiple measurements of the outcome, both pre and post the intervention/exposure? Outcomes (self-efficacy, task efficacy, outcome expectancy, and intention towards vaccination) | Yes | Yes | Is there congruity between the research methodology and the interpretation of results? | Yes | Are the processes of gathering and summarizing the evidence described? | Yes |
| Q6 | Were valid methods used for the identification of the condition? | Yes | Yes | Do you consider this account to be a narrative? | Yes | Yes | Yes | Are costs and outcomes valued credibly? | Yes | Were strategies to deal with confounding factors stated? | Not Applicable | Were the outcomes of participants included in any comparisons measured in the same way? | Yes | Yes | Is there a statement locating the researcher culturally or theoretically? | Not Applicable | Is any incongruence with the extant literature/evidence logically defended? | Not Applicable |
| Q7 | Was the condition measured in a standard, reliable way for all participants? | Yes | Yes |  |  |  |  | Are costs and outcomes adjusted for differential timing? | Yes | Were outcomes measured in a reliable way? | Yes | Were outcomes measured in a reliable way? | Yes | Yes | Is the influence of the researcher on the research, and vice- versa, addressed? | No | Are the methods used to develop recommendations described? | Yes |
| Q8 | Was there appropriate statistical analysis? | Yes | Yes |  |  |  |  | Is there an incremental analysis of costs and consequences? | No | Was appropriate statistical analysis used? | Yes | Was follow-up complete and if not, were differences between groups in terms of their follow-up adequately described and analyzed? | Not Applicable | Yes | Are participants, and their voices, adequately represented? | Yes |  |  |
| Q9 | Was the response rate adequate, and if not, was the low response rate managed appropriately? | Not Applicable | Yes |  |  |  |  | Were sensitivity analyses conducted to investigate uncertainty in estimates of cost or consequences? | Yes |  |  | Was appropriate statistical analysis used? | Yes | Yes | Is the research ethical according to current criteria or, for recent studies, and is there evidence of ethical approval by an appropriate body? | Not Applicable |  |  |
| Q10 |  |  |  |  |  |  |  | Do study results include all issues of concern to users? | Yes |  |  |  |  |  | Do the conclusions drawn in the research report flow from the analysis, or interpretation, of the data? | Yes |  |  |
| Q11 |  |  |  |  |  |  |  | Are the results generalizable to the setting of interest in the review? | Yes |  |  |  |  |  |  |  |  |  |
| **Overall score (%)** |  | **87,5** | **100** |  | **100** | **100** | **100** |  | **88,9** |  | **71,4** |  | **100** | **100** |  | **85,7** |  | **100** |

**Supplementary Table 2**. Critical quality appraisal.

Quality thresholds were set as follows: scores below 55% were classified as “poor,” 55%-70% as “fair,” and above 70% as “good methodological quality.”

*For the tool, costs were not considered in economic terms but in human terms.

**References**

[1] "JBI, Critical Appraisal Tools." JBI. <https://jbi.global/critical-appraisal-tools> (accessed 06/06/2024).

[2] D. Vega Ocasio *et al.*, "Cholera Outbreak - Haiti, September 2022-January 2023," *MMWR Morb Mortal Wkly Rep,* vol. 72, no. 2, pp. 21-25, Jan 13 2023, doi: 10.15585/mmwr.mm7202a1.

[3] B. Babakura *et al.*, "The challenges of insecurity on implementing vaccination campaign and its effect on measles elimination and control efforts: A case study of 2017/18 measles campaign in Borno state, Nigeria," *Vaccine,* vol. 39 Suppl 3, pp. C66-C75, Nov 17 2021, doi: 10.1016/j.vaccine.2021.01.024.

[4] E. Cambaza, E. Mongo, E. Anapakala, R. Nhambire, J. Singo, and E. Machava, "Outbreak of Cholera Due to Cyclone Kenneth in Northern Mozambique, 2019," *Int J Environ Res Public Health,* vol. 16, no. 16, Aug 15 2019, doi: 10.3390/ijerph16162925.

[5] J. R. Aceng *et al.*, "Uganda's experience in Ebola virus disease outbreak preparedness, 2018-2019," *Global Health,* vol. 16, no. 1, p. 24, Mar 19 2020, doi: 10.1186/s12992-020-00548-5.

[6] V. Aggrawal *et al.*, "Disease surveillance during a large religious mass gathering in India: The Prayagraj Kumbh 2019 experience," *Int J Infect Dis,* vol. 101, pp. 167-173, Dec 2020, doi: 10.1016/j.ijid.2020.09.1424.

[7] J. Havumaki, R. Meza, C. R. Phares, K. Date, and M. C. Eisenberg, "Comparing alternative cholera vaccination strategies in Maela refugee camp: using a transmission model in public health practice," *BMC Infect Dis,* vol. 19, no. 1, p. 1075, Dec 21 2019, doi: 10.1186/s12879-019-4688-6.

[8] G. J. Kost, A. K. Fuzery, L. K. R. Caratao, S. Tinsay, A. Zadran, and A. P. Ybanez, "Using geographic rescue time contours, point-of-care strategies, and spatial care paths to prepare island communities for global warming, rising oceans, and weather disasters," *Int J Health Geogr,* vol. 22, no. 1, p. 38, Dec 20 2023, doi: 10.1186/s12942-023-00359-y.

[9] J. C. Ugwuoke, F. O. Talabi, O. Adelabu, B. O. Sanusi, V. C. Gever, and C. Onuora, "Expanding the boundaries of vaccine discourse: impact of visual illustrations communication intervention on intention towards COVID-19 vaccination among victims of insecurity in Nigeria," *Hum Vaccin Immunother,* vol. 17, no. 10, pp. 3450-3456, Oct 3 2021, doi: 10.1080/21645515.2021.1886558.

[10] O. Oladeji *et al.*, "Integrating immunisation services into nutrition sites to improve immunisation status of internally displaced persons' children living in Bentiu protection of civilian site, South Sudan," *Pan Afr Med J,* vol. 32, p. 28, 2019, doi: 10.11604/pamj.2019.32.28.15464.

[11] L. S. Anam, M. M. Badi, M. A. Assada, and A. A. Al Serouri, "Evaluation of Two Malaria Surveillance Systems in Yemen Using Updated CDC Guidelines: Lessons Learned and Future Perspectives," *Inquiry,* vol. 56, p. 46958019880736, Jan-Dec 2019, doi: 10.1177/0046958019880736.

[12] A. R. Ario *et al.*, "The logic model for Uganda's health sector preparedness for public health threats and emergencies," *Glob Health Action,* vol. 12, no. 1, p. 1664103, 2019, doi: 10.1080/16549716.2019.1664103.
